# Introduction of Criterion-Based Audit of Postpartum Hemorrhage in a University Hospital in Eastern Ethiopia: Implementation and Considerations

**DOI:** 10.3390/ijerph17249281

**Published:** 2020-12-11

**Authors:** Abera Kenay Tura, Yasmin Aboul-Ela, Sagni Girma Fage, Semir Sultan Ahmed, Sicco Scherjon, Jos van Roosmalen, Jelle Stekelenburg, Joost Zwart, Thomas van den Akker

**Affiliations:** 1 School of Nursing and Midwifery, College of Health and Medical Sciences, Haramaya University, P.O. Box 235 Harar, Ethiopia; giruu06@gmail.com; 2 Department of Obstetrics and Gynaecology, University Medical Centre Groningen, 9700 RB Groningen, The Netherlands; s.a.scherjon@umcg.nl; 3 Department of Obstetrics and Gynaecology, Leiden University Medical Centre, 2300 RC Leiden, The Netherlands; Yasmin.aboulela@outlook.com (Y.A.-E.); J.J.M.van_Roosmalen@lumc.nl (J.v.R.); T.H.van_den_Akker@lumc.nl (T.v.d.A.); 4 Department of Obstetrics and Gynaecology, Hiwot Fana Specialized University Hospital, P.O. Box 235 Harar, Ethiopia; sultan.semir@gmail.com; 5 Athena Institute, Vrije Universiteit Amsterdam, 1081 HV Amsterdam, The Netherlands; 6 Department of Health Sciences, Global Health, University Medical Centre Groningen, 9700 AD Groningen, The Netherlands; jelle.stekelenburg@online.nl; 7 Department of Obstetrics and Gynaecology, Leeuwarden Medical Centre, 8934 AD Leeuwarden, The Netherlands; 8 Department of Obstetrics and Gynaecology, Deventer Ziekenhuis, 7416 SE Deventer, The Netherlands; j.zwart@dz.nl

**Keywords:** criterion-based audit, postpartum hemorrhage, Ethiopia

## Abstract

With postpartum hemorrhage (PPH) continuing to be the leading cause of maternal mortality in most low-resource settings, an audit of the quality of care in health facilities is essential. The purpose of this study was to identify areas of substandard care and establish recommendations for the management of PPH in Hiwot Fana Specialized University Hospital, eastern Ethiopia. Using standard criteria (*n* = 8) adapted to the local hospital setting, we audited 45 women with PPH admitted from August 2018 to March 2019. Four criteria were agreed as being low: IV line-setup (32 women, 71.1%), accurate postpartum vital sign monitoring (23 women, 51.1%), performing typing and cross-matching (22 women, 48.9%), and fluid intake/output chart maintenance (6 women, 13.3%). In only 3 out of 45 women (6.7%), all eight standard criteria were met. Deficiencies in the case of note documentation and clinical monitoring, non-availability of medical resources and blood for transfusion, as well as delays in clinical management were identified. The audit created awareness, resulting in self-reflection of current practice and promoted a sense of responsibility to improve care among hospital staff. Locally appropriate recommendations and an intervention plan based on available resources were formulated.

## 1. Introduction

The Ethiopian Maternal Mortality Ration (MMR) is among the highest in the world, with an estimated 401 maternal deaths per 100,000 live births [1]. With this high MMR, Ethiopia is one of the ten countries which together account for 59% of all maternal deaths worldwide [2]. Direct obstetric causes—obstetric hemorrhage, hypertensive disorders of pregnancy, and sepsis—are the leading causes of maternal death in Ethiopia, contributing to more than 80% of all deaths [3].

Postpartum hemorrhage (PPH), defined as vaginal bleeding with blood loss of ≥500 mL within 24 h after birth, accounts for almost one-quarter of all maternal deaths globally [4]. Although knowledge of the burden of obstetric hemorrhage, its underlying causes, and prevention modalities are well established [5,6,7,8], PPH continues to be the leading cause of maternal mortality and morbidity, especially in low-resource settings. Achieving the target of reducing the global MMR to less than 70 per 100,000 live births by 2030 will therefore require interventions aiming at reducing preventable deaths from obstetric hemorrhage, among others. Given the considerably increased uptake of facility-based maternity care in Ethiopia in recent years [9], improving quality of care at the facility level is necessary to contribute to achieving this target.

In 2016, the initiative of improving obstetric care to women living in eastern Ethiopia was started in Hiwot Fana Specialized University Hospital (HFSUH) to identify maternal morbidity and mortality [10]. Hypertensive disorders of pregnancy, obstetric hemorrhage, and sepsis were identified as the major underlying conditions among 622 women with life-threatening complications [10,11]. Following these findings, the Averting Maternal And Neonatal Morbidity And Mortality through obstetric Audit (AMAN-MAMA) project was introduced in HFSUH to improve care practices and work towards improving outcome [12]. The AMAN-MAMA project involves performing maternal and perinatal death reviews and a criterion-based audit of obstetric complications to upgrade the quality of hospital care. Implementing an audit of PPH was one important element of the project.

Since most deaths due to PPH occur within the first 24 h after birth and can be avoided through timely and appropriate management [13], an audit of provided care is essential to design and implement appropriate interventions. Criterion-based audit (CBA) is a systematic and critical analysis that seeks to improve quality of care through review of cases against explicit criteria and use findings to modify practice as necessary [14]. The importance of CBA and its effectiveness in improving outcome of care has previously been demonstrated, also in resource-limited settings [15,16]. In addition to improving the quality of obstetric care, CBA was found to enable health care providers to reflect upon their clinical practice, explore their working environment, and give them the opportunity to think of locally applicable recommendations [17]. CBA of management of PPH has been introduced in other low- and middle-income countries such as Malawi [18], Nigeria [19], and Namibia [20] and was associated with improved outcome. In this paper, introduction of the audit process, findings of the baseline assessment, and recommendations and planned interventions are reported.

## 2. Materials and Methods

### 2.1. Study Setting and Period

This baseline study took place from 1 January 2019 to 31 March 2019 in HFSUH, a major referral center for hospitals located in eastern Ethiopia. HFSUH receives patients from district hospitals which are linked with a large number of health centers in the region. The majority of women with severe obstetric complications are referred to HFSUH due to shortage of blood for transfusion, equipment, and treatment options in nearby health centers and district hospitals [10]. Cases being referred from the nearby district facilities, located from 20 to 75 KM away, were also included. Although these facilities are also equipped with some resources to manage PPH, including injectable uterotonics, most of them refer women with PPH due to lack of blood for transfusion.

### 2.2. Study Design and Population

We implemented CBA based on the typical audit cycle, consisting of five steps: (1) establish criteria for best practice, (2) measure current practice, (3) feedback findings and set local standards, (4) implement changes, (5) re-evaluate practice and feedback [15]. In this paper, we report steps 1 to 3 of the clinical audit cycle.

#### 2.2.1. Step 1: Establishing Criteria for Best Practice

Standard of care criteria for managing PPH were adapted from the national management protocol on selected obstetric topics by the Federal Ministry of Health of Ethiopia [21] and the literature [15,22]. Eight criteria were established through two consultative meetings with obstetricians and midwives in the hospital, considering local conditions (Box 1).

Box 1 **Standards of care for management of postpartum hemorrhage in HFSUH, 2019.**

1. Patient history should be documented in case notes on admission, and include age, parity, and complications in current and or previous pregnancies.
2. General clinical state on admission should be recorded: pulse, blood pressure, temperature.
3. Intravenous (IV) line should be set up and IV fluids (crystalloids or colloids) given continuously until cross-matched blood is available.
4. Typing and cross-matching is always performed.
5. The patient’s hemoglobin level is measured.
6. Vital signs (pulse and blood pressure) are monitored at least half hourly for 2 h postpartum (or upon admission in the case of referrals).
7. A fluid intake/output chart (IV fluid and urine output) is maintained.
8. Oxytocic drugs are administered.

#### 2.2.2. Step 2: Measure Current Practice

All women with PPH admitted between August 2018 and 31 March 2019 were identified by YA and verified by a senior attending clinician (SSA). Data on sociodemographic and obstetric conditions, obstetric complications, management of PPH, and a section for audit using the eight criteria were extracted. The audit was executed based on the assumption of the “*not documented is not done*” principle. For criterion 4 (typing and cross-matching), both the blood typing and cross-matching had to be performed in order to meet the criterion. For criterion 6 (vital signs are monitored at least half-hourly for 2 h postpartum), four readings had to be performed immediately after birth in HFSUH or on admission in the case of a referral. Having vital sign readings that were more than 30 min apart meant that this criterion was not met. Each case of PPH was independently assessed by three members, consisting of a senior obstetrician from the hospital (SSA), a senior nurse midwife, and a final year medical student from Leiden University Medical Centre (YA). In cases of disagreement between the assessors, the majority ruled. In cases where there were documentation gaps or conflicting data entries, women’s case files were re-examined and the final decision was made by YA in consultation with the AMAN-MANA project leader (AKT).

#### 2.2.3. Step 3: Feedback Findings and Set Local Standards

After the baseline assessment, a seminar was organized, which was attended by the chief clinical director, and the majority of consultant obstetricians, residents, midwives, and medical interns, in order to communicate areas of substandard care and propose interventions.

### 2.3. Sample Size Consideration and Sampling

Sample size calculations were made based upon the assumption that the current standard of care (meeting each criteria) was 33%, a power of 80%, and significance level of *p* < 0.05. The aim was to achieve a 30% improvement post intervention, resulting in 43% of cases meeting standard care in the re-audit group. In order to meet these goals, a calculated total sample of 86 (43 baseline group, 43 re-audit group) was required.

### 2.4. Statistical Analyses

All data were entered into EpiData 3.1 and exported to SPSS 24 for analysis. Continuous and categorical data were described using mean (standard deviation) and frequency (percentage), respectively. We presented attainment of audit criteria separately for women with PPH referred from other facilities and in-hospital ones. Differences in meeting the audit criteria between women who gave birth in the hospital and those who reached after delivery were measured using a Chi-square test. In addition, we used tables to describe areas of substandard care and recommendations.

### 2.5. Ethics

The AMAN-MAMA project has been approved by the Institutional Health Research Ethics Review Committee (IHRERC) of the College of Health and Medical Sciences, Haramaya University in Ethiopia (Ref no: IHRERC/170/2018). This study has been conducted in accordance with the Helsinki Declaration of ethical principles for medical research involving human subjects [23]. Prior to data collection, informed consent had been obtained from the head of the hospital, and of the obstetrics and gynecology department. As this was a review of routinely completed patients’ case notes, informed consent from patients was not required. In order to mask the identity of the patients, personal and medical information were entered anonymously and no identifiable data were collected.

## 3. Results

A total of 45 women with PPH were included in this study. The mean maternal age was 25.6 (SD ± 5.76) years, ranging from 16 to 40 years. A majority of the women gave birth vaginally (38; 84.4%) and were referred from other facilities (32; 71.1%). Half had not attended any antenatal care (23; 51.1%). The most common causes of PPH were uterine atony (55.6%) followed by retained placenta (28.9%). In 24.4% of PPH cases, the cause of bleeding was not specified or documented (Table 1).

### Audit of Attainment of Standard Criteria

Meeting the standard criteria ranged from as high as 100% (45/45) for documenting patient history and recording general status on admission to as low as 13.3% (6/45) for maintaining fluid intake and output charts. Differences were noticed between women who gave birth in HFSUH and those who were referred from the nearby institutions, especially with regard to the involvement of senior obstetricians (*p* = 0.01) and administration of uterotonics (*p* = 0.01). Both of these aspects were present less often among referred women who generally arrived with life-threatening complications. However, compared to women who gave birth at HFSUH, typing and cross-matching was higher among those referred after delivery (*p* = 0.02). We found that only 3.7% (1/27) of referred women and 11.1% (2/18) of those in-hospital fulfilled all eight standard care criteria for PPH management (Table 2).

After presenting the baseline findings to staff of the obstetrics and gynecology department, a seminar was organized to move from numbers to solutions. During this meeting, the chief clinical director, consultant obstetricians, senior residents, midwives, and our research team discussed current practice, the reasons beyond the numbers, struggles staff are facing, and identified areas of substandard care. Poor documentation and chart keeping, lack of PPH management documentation, lack of equipment and supplies, shortage of blood for transfusion, and lack of clarity about roles and responsibilities in PPH management were identified (Table 3).

After identification of substandard care, recommendations from similar interventions in other low-resource settings were presented and discussed, from which locally applicable recommendations were formulated. A decision was made to establish a PPH response team (with clear roles and responsibilities), develop a PPH kit and guidelines, and use flow charts and templates for documentation. In addition, establishing a mini-blood bank in the unit, purchasing quality vital sign monitoring equipment, using simulation trainings and drills, and initiating monthly follow up for measuring progress to corrective actions were recommended (Table 4).

## 4. Discussion

In this paper, we presented the introduction of CBA of the management of PPH in a university hospital in eastern Ethiopia and the lessons learned from the baseline assessment. This study shows that using an audit can be a useful tool and a first step in initiating the process of improving quality of care at hospital level. The introduction of the audit created an opportunity to reflect on current practice, increased awareness, and motivated health care workers in HFSUH to be in charge of improving future practice [25,26,27].

Four criteria of our CBA were below standards: vital signs monitoring, IV-line setup and fluid administration, typing and cross-matching, and maintenance of a fluid intake/output chart. These findings are in line with findings in other low-income settings [17,19]. In a study conducted in 2008 in Malawi, attainment of typing and cross-matching, measuring hemoglobin levels, vital signs monitoring, and fluid intake/output chart maintenance also scored low [17]. Low IV-line setup and fluid administration could be explained by poor documentation in patient files. During the presentation of findings and feedback, it appeared that IV setup was a routine procedure in the case of PPH and fluid availability was not an issue; it was reported that these, although not documented, are part of routine care. In a study in Uganda, sub-optimal management was observed in clinical monitoring and hourly urinary output measurements in the baseline assessment of the obstetric hemorrhage group [19]. Despite challenges faced in these similar studies, both concluded that introducing CBA created an entry-point for identifying non-clinical causes of poor performance, which, when addressed, may lead to significant improvements in care [17,19].

Significant differences were observed in meeting audit criteria between women who gave birth in the hospital and those who reached facilities after delivery in three criteria: typing and cross-matching blood, administration of uterotonics, and the involvement of senior staff in the case management of severe PPH. While senior involvement and administration of uterotonics were more likely among women who gave birth in the hospital, cross-matching and typing of blood was higher among women who reached the hospital after delivery. Since the majority of women were referred to hospitals because of lack of blood in the peripheral district hospitals, receiving clinicians will be more likely to initiate the process for preparing the women for blood transfusion. In addition, given that women reaching hospitals after delivery were more likely with complications, it is worrisome that involvement of senior consultants in their management is minimal. Further audit of whether this is related to documentation or the presence of limited participation should be done.

Although maternity care in Ethiopia is officially free and should be accessible to all women [28], chronic shortage of drugs and supplies is another major challenge. Medical supplies or medicines that were not available were put on an order sheet and given to relatives to be purchased in a pharmacy, causing significant delay in management, reducing the quality of care, and increasing the risk of obstetric complications. To prevent this, a PPH kit containing all essential supplies and medication needed in an emergency case of PPH needs to be available as per international recommendations [29,30].

Lack of teamwork was identified to contribute to delays in management of PPH. In addition, lack of clarity on some of the tasks in the labor ward was mentioned. As a majority of the ward activities are performed by medical interns, the role of midwives, especially in documentation, was questioned. Given that medical interns are juniors and the involvement of midwives in the documentation is minimal, there is a need to implement obstetric care as teamwork, by which medical interns, midwives, residents, and consultants know and discharge their responsibilities. To facilitate this, a PPH response team was established, reiterating that *readiness*, *recognition,* multidisciplinary *response*, and *reporting* are essential [29]. For the PPH response team, continuous monthly drills are planned on a regular basis to strengthen teamwork in PPH management and assess team functionality. In addition, by displaying patient admission flow charts in the labor wards, we aim to structure the process of admissions by clarifying the responsibilities of interns, midwives, residents, and senior obstetricians. It was also agreed to put posters displaying summarized steps of PPH management in all rooms of the labor ward [17,19].

The last challenge observed was availability of blood. A regional blood bank exists in Harar and is available for requesting blood. Due to insufficient availability of blood, however, delay in the management of PPH occurred and transfusion requests by physicians were often not met. This leaves women with PPH in a severe state of anemia and in some cases, hypovolemic shock. Given that large numbers of women with PPH reach HFSUH in a severely compromised state due to a poorly functioning referral system and transport delays, initiating immediate obstetric care is further challenged due to blood shortage. In order to increase blood availability, a mini-blood bank is planned to be established in the obstetric department.

The noteworthy outcome of our study is that it built our capacity to work together as a team, promoting ownership and responsibility, and gave the opportunity to critically reflect on current practice. During audit meetings, there was a high attendance and we managed to involve all levels of hospital staff during each step of the process. Continuous emphasis was put on the idea of “*no shame, no blame*” through allowing all attendees to exchange ideas on how to move forward together without blaming. Meetings with staff of the department of obstetrics and gynecology gradually grew from hesitancy, self-defense, and blamings to self-reflections and active involvement in finding solutions [12,31,32,33]. The initial meetings were characterized by defensive feedback by the staff: repeatedly complaining about lack of resources by the staff and lack of efficient use of resources by the head of the hospital and other officials [12]. However, in the process, we observed a change in attitude, moving from heated discussions towards sitting around to come to an agreement in a peaceful manner.

As far as we know, this is the first criterion-based audit study on the management of PPH in Ethiopia and therefore, could be the first step to create change in the hospital and beyond. Due to underreporting, incorrect documentation of diagnosis, and mistakes during registering medical record numbers, there was high number of exclusions or missed files. Accessing case files was also difficult due to non-digital archiving of hospital files. Often, women’s case files selected from registers could not be found in the archive which meant that some cases of PPH might be missed.

Factors that contributed to low attainment of certain criteria and substandard care were identified: poor documentation and chart keeping, inadequate clinical monitoring, lack of availability of medical resources and supplies, limited availability of blood for transfusion, and delay in the management of obstetric hemorrhage. In order to address this problem, a PPH follow-up chart template for vital signs monitoring and fluid input/output recordings and PPH management checklists were designed and printed for use. With regard to vital signs monitoring, substandard care was related to the issue of responsibility and lack of well-functioning blood pressure cuffs. In HFSUH, medical interns are the obstetric front-liners, providing maternity care to large numbers of women. Change should start by giving them good instructions about their tasks, including close follow-up of patients with specific attention to monitoring of vital signs and fluid intake/output.

The strength of our study included the strong participation of local stakeholders (obstetricians and gynecologists, midwives, and hospital managers) from the onset of the study in the process of adapting the evaluation criteria and continuously participating and contributing to meetings during presentation of the baseline assessment, identification of substandard care, and proposing interventions for improvement. Our study also has some limitations. First, although active participation of staff was achieved, the fact that the research was led by external researchers may result in the research not being entirely embedded into the hospital system. Second, poor documentation of medical records in general and PPH management in particular causes difficultly in differentiating whether there were problems in implementing the criteria or lack of proper documentation. As we followed the principle of “not documented not done”, consensus was reached about the importance of documentation in patient monitoring and identifying deficiencies in the care.

## 5. Conclusions

In this paper, we presented the introduction process of CBA in eastern Ethiopia, as well as findings from a baseline assessment as an element of the audit cycle. The first three steps of the audit cycle—establishing criteria for best practice, measurement of current practice, and feedback findings and setting local standards—were addressed. The successful completion of this first phase lays the foundation for the remaining steps. Introduction of the audit was found to be an effective educational tool and a mechanism for initiating first steps towards improving the quality of obstetric care [34,35]. We found that the current quality of PPH management did not meet the majority of the standard criteria. Major areas of substandard care have been identified and locally appropriate recommendations were made. Moving towards the next phase, implementing the proposed interventions over a period of several months, we intend to perform a re-audit at a later stage to assess the impact of the audit on the management of PPH and overall improvement in quality of care.

## Figures and Tables

**Table 1:** Sociodemographic and obstetric conditions of women with postpartum hemorrhage (PPH) in a university hospital in eastern Ethiopia, 2020.

Variables	*n*	%
**Parity**		
0	12	26.7
1–4	18	40.0
>4	15	33.3
**Received antenatal care (at least one)**		
Yes	22	48.9
No	23	51.1
**Referred from other facility**		
Yes	33	73.3
No	12	26.7
**Birth location**		
HFSU	18	40.0
District hospital	5	11.1
Health center	9	20.0
Home	8	17.8
En route	5	11.1
Causes of PPH *		
Uterine atony	25	55.6
Retained placenta	13	28.9
Uterine atony and retained placenta	2	4.4
Cervical laceration	2	4.4
Tear of birth canal	4	8.9
Coagulopathy	1	2.2
Not specified	11	24.4

SD—standard deviation; HFSUH—Hiwot Fana Specialized University Hospital; * Total exceeds 45 since some women with PPH had more than one cause.

**Table 2:** Attainment of standard criteria for PPH management in HFSUH, 2019.

SN	Standard of Care	All PPH Cases (n = 45)	Came after Birth (n = 27)	In-HFSUH Cases (n = 18)	*p*-Value *
1.	Documented patients’ history on admission	45 (100%)	27 (100)	18 (100%)	-
2.	Recorded general clinical state on admission	44 (97.8%)	26 (96.3)	18 (100%)	-
3.	Intravenous line set up and fluids given continuously	32 (71.1%)	23 (85.2)	11 (61.1%)	0.07
4.	Typing and cross-match performed	22 (48.9%)	17 (63.0)	5 (27.8%)	0.02
5.	Hemoglobin or hematocrit established	43 (95.6%)	26 (96.3)	17 (94.4%)	0.77
6.	Vital signs monitored at least half hourly for 2 h postpartum	23 (51.1%)	14 (51.9)	8 (44.4%)	0.47
7.	Fluid intake/output chart maintained	6 (13.3%)	3 (11.1)	3 (16.7%)	0.59
8.	Oxytocic drugs administered	30 (66.7%)	14 (51.9)	16 (88.9%)	0.01
9.	Senior obstetrician involved in the care ^+^	18/29(62.1)	11/21 (52.4)	7/8 (87.5)	0.01
	Meeting all standard criteria (1 to 8)	3 (6.7%)	1(3.7)	2 (11.1%)	0.33

IV—intravenous; PPH—postpartum hemorrhage; HFSUH—Hiwot Fana Specialized University Hospital; * Chi-square test (between those who gave birth in the hospital and who came after birth); ^+^ Among women with severe PPH (*n* = 29).

**Table 3:** Substandard care factors in management of PPH in HFSUH, eastern Ethiopia, 2019.

SN	Sub-Standard Care Factors
1.	Poor documentation and chart keeping (including vital sign recordings, partograph maintenance, birth summary, discharge summary, input/output charts)
2.	Lack of documentation of management of postpartum hemorrhage
3.	Incomplete vital sign monitoring immediately postpartum
4.	Lack of and poor quality of available blood pressure cuffs
5.	Lack of blood for transfusion
6.	Poor referral system causing delay and/or resulting in severe anemia and hypovolemic shock
7.	Delay in initiating assessment of emergency cases
8.	Lack of medical resources and supplies (medical equipment, medication)
9.	Not specifying cause of PPH, especially among referred patients
10.	Responsibilities of medical staff not clear (especially for medical interns)

PPH—postpartum hemorrhage.

**Table 4:** Recommendations for improving PPH management in HFSUH, 2019.

SN	Recommendations for Implementation
1.	A PPH response team consisting of one obstetrician, one resident, three interns, and four midwives should be established, members need to be identified, rotation schedules made, and drills performed on regular basis.
2.	A PPH kit (containing medications, supplies, checklists, and instruction cards) will be created and kept in the labor ward; PPH team responsible for stock out and completeness at all times.
3.	PPH guidelines specifically for HFSUH need to be finalized and posters with standard PPH management criteria/steps to be put up in triage, the labor ward, and maternity ward.
4.	PartoMa pocket guidelines for management of obstetric complications, as implemented in a study by Maaløe N et al. in Zanzibar [24], to be contextualized for use in HFSUH.
5.	Documentation should be improved by implementing: A PPH follow up chart (vital signs, blood loss, uterine status, fluid intake/output); A PPH management checklist to be filled in after management and stabilization of patient, name of staff involved to be documented.
6.	Sufficient blood should be made available through establishing a mini-blood bank for the maternity ward.
7.	Punchers to be purchased to reduce incompleteness of patients’ cards and prevent loose sheets from getting lost.
8.	Thirteen good quality blood pressure cuffs to be purchased, two for each room in the labor ward, one for triage.
9.	An admission patient flow chart will be made and put up in the labor ward and triage, which includes who is responsible for what part of management in case of a new PPH admission.
10.	Training with simulations for residents on B-lynch standard procedure and uterine artery ligation will be organized; PPH team responsible for organization.
11.	Monthly presentation and meeting to discuss on what went well, what can be done better, and what actions to be taken to reach set objectives.

PPH—postpartum hemorrhage; HFSUH—Hiwot Fana Specialized University Hospital.
